# Taking Decisions Too Seriously: Why Maximizers Often Get Mired in Choices

**DOI:** 10.3389/fpsyg.2022.878552

**Published:** 2022-06-15

**Authors:** Mo Luan, Zhengtai Liu, Hong Li

**Affiliations:** ^1^School of Business, University of International Business and Economics, Beijing, China; ^2^Department of Psychology, Tsinghua University, Beijing, China

**Keywords:** maximizers, satisficers, assortment size, perceived importance, decision making

## Abstract

Maximizing is a topic that has received significant attention from researchers and corporate organizations alike. Although extensive previous research has explored how maximizers behave in a decision scenario, a fundamental question remains about why they prefer a larger assortment regardless of whether the decisions are important or not. This study attempts to explore the underlying mechanism of this phenomenon. Four surveys were conducted, and participants from Mturk or Credamo online platforms were recruited (*N* = 922). The maximizing tendency was measured by either maximization scale or maximizing tendency scale, and perceived importance and preference for a large assortment were measured in different decision scenarios. Across four studies, we find that maximizers perceive the same decision as more important than satisficers (Study 1), and perceived importance serves as the mechanism underlying the maximizers’ preference for a large assortment (Study 2). In other words, in maximizers’ perceptions and interpretations, even seemingly trivial decisions are important enough to spend great effort on a large assortment. We additionally identified a boundary condition for the effect – cost salience (Studies 3a and 3b). These findings illustrate a pioneering empirical exploration of the difference in the way maximizers and satisficers perceive their decision importance and the reason for maximizers’ preference for a large assortment.

## Introduction

Both conventional wisdom and research propose that individuals should only search for a large assortment when the decision contents are important (Chaiken and Maheswaran, 1994; Sela and Berger, 2012). In other words, it is reasonable that people expend more effort on important decisions. However, maximizers (i.e., decision-makers who strive for the best possible option) seem to be the exception: they get mired in choices not only when the decision is important but also when the decision seems trivial. Regardless of whether they buy a car or choose a chocolate bar, maximizers prefer retailers that offer large assortments in order to obtain the best possible option, even though the large assortment comes with a salient cost (Chowdhury et al., 2009; Brannon and Soltwisch, 2017). For example, maximizers are more likely to drive 25 min to a grand superstore with 25 different alternatives for cleaning supplies, rather than visit the nearest grocery store that might have just four alternatives (Dar-Nimrod et al., 2009). Although previous research has predominantly focused on exploring how maximizers behave in a decision scenario (Schwartz et al., 2002; Ma and Roese, 2014), a fundamental question remains about why they prefer a larger assortment regardless of whether the decisions are important or not.

Many argue that individuals perceive the importance of a decision based on his/her own perception and interpretation (Posavac and Herzenstein, 2003). In other words, a decision (e.g., purchasing a sweater) can be perceived as important or unimportant subjectively. This raises the question of whether maximizers perceive a decision as more important than their counterparts (i.e., satisficers, decision-makers who settle for a good enough option), which could result in the choice of a store that is cost salient but that has a large assortment. In particular, whether the seemingly trivial decisions for others are indeed important for maximizers? In this study, we are particularly concerned with why maximizers prefer a larger assortment regardless of whether the decisions are important or not. In the following paragraphs, the literature background will be introduced, and hypotheses will be developed.

The term “maximizer” was first proposed by Schwartz et al. (2002) who used it to refer to those who search for large assortments and seek the best choice. In contrast, the term “satisficer” is used to refer to those who search for relatively small assortments and settle down for a good enough choice. Further research found that the decision-making styles remained stable among different contexts (Kokkoris, 2019; Moyano-Díaz and Mendoza-Llanos, 2021). Regardless of the context or importance, whether it be seeking a job, selecting a course, shopping for dish soap, or choosing an ice-cream flavor, maximizers prefer a larger assortment than satisficers (Iyengar et al., 2006; Chowdhury et al., 2009; Dar-Nimrod et al., 2009; Carrillat et al., 2011; Cheek and Schwartz, 2016; Luan and Li, 2017). However, although previous research revealed the domain-spanning preference for a large assortment, few studies have explored the mechanism behind this phenomenon.

Notably, a typical type of decision is purchasing decisions. In these decisions, the choice of assortment (i.e., the number of options in a single product category) (Broniarczyk, 2008; Goodman and Malkoc, 2012) can be conceptualized in a two-stage hierarchical fashion. In the first stage, people choose a store to visit. In the second stage, they choose a certain product in the store (Broniarczyk, 2008; Goodman and Malkoc, 2012). Thus, the assortment size that decision-makers can choose from is largely determined by the store they visit. In this study, we concentrate on the first stage—decision makers’ preference for stores with different assortment sizes. Specifically, we focus on why maximizers are more attracted to retailers that offer larger assortments.

Decision importance describes how consequential a decision is (Posavac and Herzenstein, 2003). A decision is more important when its consequences have more impact than those of other decisions (Krijnen et al., 2015). Previous research has found that decision importance is a critical factor that influences people’s preferences and decision processes. When decisions are important, people are motivated to obtain a good choice; thus, they expend more effort and search for more alternatives (Chaiken and Maheswaran, 1994; Sela and Berger, 2012). However, importance is relative and differs from person to person. The same decision can be perceived as important or not, based on the decision makers’ subjective perception and interpretation (Posavac and Herzenstein, 2003). Individuals allocate effort and desire to a certain assortment size according to their perceived importance of the decision (Payne et al., 1993). When decision-makers perceive the decisions as trivial, they allocate less effort and search for a limited number of options; otherwise, they allocate more effort and choose a larger assortment.

How do maximizers and satisficers perceive the importance of a decision differently? To the best of our knowledge, less research has provided empirical evidence for this question. However, from the fact that maximizers get stuck in decisions, especially minor ones, we infer that a seemingly trivial decision for others might indeed be perceived as important by maximizers. In other words, maximizers do not really get stuck in (what appear to be) trivial decisions for themselves because they perceive the decision as important, although others may view it differently (for example, others might perceive purchasing soap as trivial, but maximizers might perceive it as important). In this sense, it is reasonable that maximizers expend great effort and seek a large assortment because the decisions are important to their perceptions. Thus, we propose that the perceived importance of a decision is a critical factor that can explain maximizers’ preference for a large assortment size. Accordingly, we hypothesize the following:

**H1:** When faced with the same decision, maximizers perceive it as more important than satisficers do.

**H2:** Perceived importance mediates the relationship between maximizing and preference for a large assortment.

One basic assumption of this study is that a large assortment often comes with a cost (Chernev et al., 2015). For example, large retailers have a wider scope of coverage than small ones; thus, on average, people need to expend more effort (spend more time) to visit the stores offering larger assortments. Based on H2, perceived importance drives maximizers to choose stores that have a large assortment, despite the additional effort to visit them. However, a key question to consider is what if a large assortment does not come with a salient cost. Previous research suggests that without explicit cost information, people may not be intuitively aware of the cost of a large assortment choice (for example, decision-makers do not automatically consider the cost to reach a store with a large assortment) (Iyengar and Lepper, 2000; Goodman and Malkoc, 2012). Researchers have demonstrated that when making an assortment decision, focusing on the cost (effort expended or difficulty encountered) associated with the decision process can decrease the preference for large assortments. However, without considering cost, people are generally attracted to large assortments (Goodman and Malkoc, 2012). Therefore, we propose that when a large assortment does not come with a salient cost, people generally prefer a large assortment regardless of perceived importance. Accordingly, we hypothesize the following:

**H3a:** When a large assortment comes with a salient cost, perceived importance leads to a greater preference for a large assortment.

**H3b:** When a large assortment does not come with a salient cost, perceived importance does not significantly predict participants’ preference for a large assortment.

**FIGURE 1:**
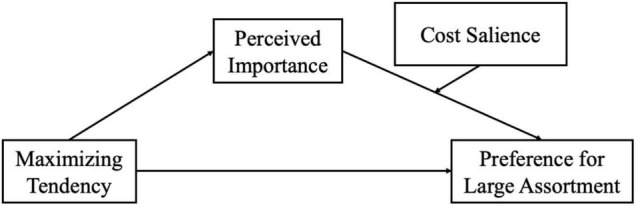
Conceptual framework—moderated mediation model.

Three studies were conducted to support an overall moderated mediation model (Figure 1). Study 1 tested the basic proposition that maximizers perceive a decision as more important than satisficers (H1). Study 2 confirmed that perceived importance mediates the relationship between maximizing and preference for a large assortment (H2). Studies 3a and 3b manipulated the cost salience of different choices and examined the moderating role of cost salience (H3) and the overall moderated mediation model.

## Study 1

Study 1 tested our basic prediction that when faced with the same decision, maximizers perceive it as more important compared to satisficers (H1). In this study, maximizers and satisficers were measured using the maximization scale (Schwartz et al., 2002). Eleven products were chosen as the decision stimuli.

### Method

#### Participants

Recruitment was open to 202 US-based participants (93 females, 109 males, *M*_*age*_ = 33.68 years, *SD* = 10.57) from Mechanical Turk (MTurk) by Amazon.com in exchange for a small payment. No participant was excluded from the analysis.

#### Procedure and Materials

First, the participants completed the maximization scale (Schwartz et al., 2002), a widely used scale to assess individuals’ tendencies to maximize. The scale contains 13 items (for example, “I never settle for second best” and “When shopping, I have a hard time finding clothing that I really love”). Each item was rated from 1 (strongly disagree) to 7 (strongly agree). The individual scores of the 13 items in the maximization scale were averaged to create a composite maximizing score (Cronbach’s α = 0.72). Higher scores represent higher maximizing tendencies, indicating that individuals are more likely to be maximizers. Lower scores indicate that individuals are more likely to be satisficers.

After a short filter task, the participants saw 11 products (i.e., sweater/toothbrush/chocolate/smartphone/shoe/sunglasses/ice maker/shampoo/dish soap/pen/car), and were asked to rate the perceived importance of each decision (for example, “How important do you perceive buying a sweater to be?”) on 100-point scales anchored to not important at all (= 0) to very important (= 100). The 11 products were presented in random order.

### Results and Discussion

We first averaged the importance ratings from each participant for the 11 products to create an overall perceived importance score. According to the results of correlation, the maximization tendency was positively correlated with the overall perceived importance (*r* = 0.40, *p* < 0.001). Specifically, the maximization tendency was positively correlated with perceived importance when the decision targets were sweaters (*r* = 0.31, *p* < 0.001), toothbrush (*r* = 0.15, *p* = 0.041), chocolate (*r* = 0.31, *p* < 0.001), smartphone (*r* = 0.28, *p* < 0.001), shoes (*r* = 0.31, *p* < 0.001), sunglasses (*r* = 0.39, *p* < 0.001), ice maker (*r* = 0.32, *p* < 0.001), shampoo (*r* = 0.26, *p* < 0.001), dish soap (*r* = 0.31, *p* < 0.001), pen (*r* = 0.36, *p* < 0.001), and car (*r* = 0.21, *p* = 0.003). Overall, the results of Study 1 support H1—maximizers have a higher perceived importance than satisficers (refer to Table 1 for descriptive statistics of all the four studies; maximizers and satisficers in Table 1 were divided based on a medium split of the scale results).

**TABLE 1:** Descriptive statistics of all the four studies.

			Maximizers		Satisficers	
				
Study	Variable	Decision scenario	*Mean*	*SD*	*Mean*	*SD*
Study 1	Perceived importance	Sweater	61.75	30.41	50.00	23.75
		Toothbrush	61.74	32.57	56.54	30.02
		Chocolate	50.81	33.67	37.17	27.96
		Smartphone	79.17	21.25	66.96	27.12
		Shoes	72.46	22.22	61.94	24.70
		Sunglasses	57.90	30.95	42.39	24.62
		Ice maker	46.79	32.54	34.13	26.02
		Shampoo	65.28	28.28	51.81	26.67
		Dish soap	56.00	33.43	40.80	28.45
		Pen	49.19	34.52	33.96	27.21
		Car	81.35	21.62	71.64	22.12
Study 2	Perceived importance	Sweater	79.07	19.48	58.12	20.99
	Preference for large assortment	Sweater	70.69	24.01	52.91	25.86
Study 3a-cost salient condition	Perceived importance	Smartphone	88.41	18.91	83.80	17.16
	Preference for large assortment	Smartphone	67.39	32.66	55.84	34.63
Study 3a-cost not-salient condition	Perceived importance	Smartphone	87.60	15.37	80.75	21.72
	Preference for large assortment	Smartphone	72.46	30.02	60.45	39.02
Study 3b-cost salient condition	Perceived importance	Sweater	74.77	21.95	54.11	23.26
	Preference for large assortment	Sweater	79.13	26.37	50.88	35.36
Study 3b-cost not-salient condition	Perceived importance	Sweater	69.02	21.50	53.37	26.79
	Preference for large assortment	Sweater	85.52	18.84	72.80	34.71

## Study 2

Study 2 was carried out to test the mediating role of perceived importance in the relationship between maximizing and preference in the case of a large assortment. In this study, participants need to indicate their relative preference between two stores, namely, a distant store (i.e., indicating that decision-makers need to expend more effort to reach the store) with a large assortment and a nearby store with a small assortment. According to H2, we predicted that compared to satisficers, maximizers perceive the decision as more important, which subsequently leads to a preference for the large assortment. Study 2 was preregistered, and the preregistration form can be found at osf.io/yjgev.

### Method

#### Participants

Recruitment was open to 200 US-based participants from Mechanical Turk (MTurk) by Amazon.com in exchange for a small payment. Following the removal of participants who failed to pass one or more attention checks, 175 participants (76 females, 99 males, *M*_*age*_ = 33.55 years, *SD* = 10.28) remained.

#### Procedure and Materials

Participants first completed the maximization scale (Schwartz et al., 2002). At the end of the scale, an attention check was conducted, where participants were asked to select the “strongly disagree” option for a question. After a short filter task, they read a scenario about buying a sweater and were asked how important the sweater-buying decision was to them on a 100-point scale anchored from not important at all (= 0) to very important (= 100). Participants were then given two stores where they could buy the sweater. They could either (A) walk to the nearest local store (5 min away) that offered eight different types of sweaters or (B) drive to the shopping mall (40 min away) that offered 40 different types of sweaters. They were instructed to indicate their relative preference on a 100-point scale anchored at the preference for Store A (= 0) and the preference for Store B (= 100). The two stores (A and B) were presented in random order. Finally, as another attention check, the participants answered which type of product they were buying in this study. Participants’ monthly consumption levels (“How much do you spend per month (in US dollars)”) were measured as the control variable.

### Results and Discussion

#### Control Variable

We first examined whether participants’ monthly consumption levels were correlated with other variables. The results of correlation revealed that monthly consumption level did not significantly correlate with maximizing tendency (*r* = 0.07, *p* = 0.343), perceived importance (*r* = –0.01, *p* = 0.926), or preference for a large assortment (*r* = –0.07, *p* = 0.377). Thus, monthly consumption was not taken as a covariate in further analyses.

#### Perceived Importance

Linear regression was conducted to test the relationship between the maximizing tendency and the perceived importance of the sweater. As predicted, participants’ maximizing tendency positively predicted their perceived importance, *R*^2^ = 0.23, *B* = 11.44, *SE* = 1.58, β = 0.48, *t* (172) = 7.23, *p* < 0.001. Consistent with the results of Study 1, maximizers perceived the decision as more important than satisficers.

#### Preference for a Large Assortment

Another linear regression was conducted to test the relationship between the maximizing tendency and preference for the large assortment. As predicted, participants’ maximizing tendency positively predicted their preference, *R*^2^ = 0.13, *B* = 9.95, *SE* = 1.95, β = 0.36, *t* (173) = 5.10, *p* < 0.001.

#### Mediating Analysis

**FIGURE 2:**
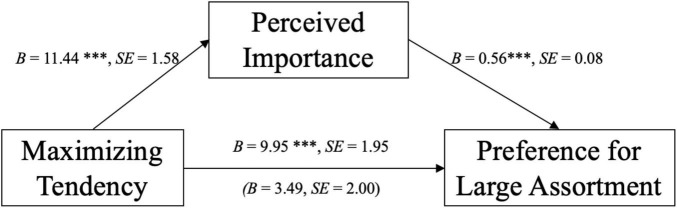
Mediation from Study 2. The value above the dashed arrow indicates the total effect of maximizing tendency on preference for a large assortment, not accounting for mediator. The value under the dashed arrow indicates the direct effect of maximizing tendency on preference for a large assortment, with perceived importance included as the mediator. ***p < 0.001.

To test our central prediction, we conducted a mediation analysis using PROCESS macro, Model 4 (Hayes, 2013) with maximizing tendency as the independent variable, preference for the large assortment as the dependent variable, and perceived importance as a mediator. A bootstrapping analysis with 5,000 iterations revealed that perceived importance mediated the relationship between maximizing tendency and preference. The 95% bias-corrected confidence interval for the indirect effect did not include zero [3.796, 9.821] and the direct effect included zero [–0.464, 7.451] (refer to Figure 2). Thus, H2 is supported, revealing that perceived importance mediates the relationship between maximizing and preference for a large assortment.

## Study 3a

Study 3a was carried out to demonstrate that the cost salience of assortment is a boundary condition of the observed effect. According to the results of Study 2, maximizers’ preferences for a large assortment are driven by their higher perceived importance. However, one basic assumption of Study 2 is that a large assortment often comes with a cost. Specifically, decision-makers need to expend more effort to reach stores with large assortments. In this study, we manipulated the cost salience of a large assortment and tested the hypothesis that perceived importance leads to a greater preference for a large assortment only when the large assortment comes with a salient cost (H3).

### Method

#### Participants

Recruitment was open to 270 US-based participants from Mechanical Turk (MTurk) by Amazon.com in exchange for a small payment. Following the removal of participants who failed to pass one or more attention checks, 244 participants (119 females, 125 males, *M*_*age*_ = 31.70 years, *SD* = 9.88) remained.

#### Procedure and Materials

Participants first completed the maximization scale (Schwartz et al., 2002). After a short filter task, they read a scenario about buying a smartphone and were asked, when making the decision, how important a smartphone was to them on a 100-point scale. The scale was anchored from not important at all (= 0) to very important (= 100). Participants were then randomly assigned to the cost salient group and the cost non-salient group. In the cost salient group, participants were then given two stores similar to Study 2. Specifically, they could either (A) walk to the nearest local store (5 min away) that offered eight different smartphones or (B) drive to the shopping mall (40 min away) that offered 40 different smartphones. In the cost non-salient group, the two stores only differed in the number of products; specifically, they could either go to store (A) that offered eight different smartphones or store (B) that offered 40 different smartphones. The participants in both groups were instructed to indicate their relative preference on a 100-point scale anchored at the preference for Store A (= 0) and the preference for Store B (= 100). Two attention checks identical to Study 2 were conducted, and participants’ monthly consumption levels were measured.

### Results and Discussion

#### Control Variable

Similar to Study 2, the monthly consumption level did not significantly correlate with maximizing tendency (*r* = –0.08, *p* = 0.230), cost salience (*r* = –0.04, *p* = 0.570), perceived importance (*r* = –0.04, *p* = 0.505), or preference for large assortments (*r* = –0.03, *p* = 0.634). Thus, monthly consumption was not taken as a covariate in further analyses.

#### Perceived Importance

As predicted, the results of linear regression revealed that participants’ maximizing tendency positively predicted their perceived importance, *R*^2^ = 0.03, *B* = 4.57, *SE* = 1.47, β = 0.20, *t* (242) = 3.10, *p* = 0.002. Consistent with the results of Studies 1 and 2, maximizers perceived the decisions as more important than satisficers.

#### Preference for a Large Assortment

Two liner regressions were conducted to test the main effect of maximizing tendency or perceived importance on preference for large assortments. Participants’ maximizing tendency positively predicted their preference for large assortments, *R*^2^ = 0.03, *B* = 7.72, *SE* = 2.79, β = 0.18, *t* (242) = 2.77, *p* = 0.006. Generally, maximizers preferred large assortments compared to satisficers. Participants’ perceived importance also positively predicted their preference for large assortments, *R*^2^ = 0.08, *B* = 0.54, *SE* = 0.12, β = 0.29, *t* (242) = 4.63, *p* < 0.001, indicating that participants prefer to search for more alternatives when they perceive the decision target as important. In addition, to test H3, we examined the moderating role of cost salience (i.e., cost salient vs. cost not salient) on the relationship between perceived importance and preference for the large assortment, using the PROCESS macro for model 1 (Hayes, 2013). The results revealed a significant cost salience—perceived importance interaction effect on preference, *F*_(1,240)_ = 4.93, *p* = 0.027. Specifically, perceived importance positively predicted participants’ preference for the large assortment when more alternatives came with a salient cost (*R*^2^ = 0.17, *B* = 0.80, *SE* = 1.53, β = 0.42, *t* (129) = 5.22, *p* < 0.001), whereas perceived importance did not significantly predict participants’ preference for a large assortment when more alternatives came with no salient cost (*R*^2^ = 0.02, *B* = 0.29, *SE* = 0.17, β = 0.16, *t* (111) = 1.65, *p* = 0.102). Thus, H3 is supported.

#### Moderated Mediation Analysis

As the moderating role of cost on the relationship between perceived importance and preference for the large assortment was supported, we further tested a moderated mediation model using PROCESS Model 14 (Hayes, 2013), with maximizing tendency as the independent variable, preference as the dependent variable, perceived importance as a mediator, and cost as the moderator (refer to Figure 1). A bootstrapping analysis with 5,000 iterations revealed that perceived importance mediated the relationship between maximizing tendency and preference under cost-salient conditions, as the 95% bias-corrected confidence interval for the indirect effect did not include zero [1.522, 5.874]. However, the mediating effect diminished under the cost non-salient condition, and the indirect effect included zero [–0.603, 3.389]. Thus, the results indicated that the mediating effect of perceived importance only existed when large assortments came with a salient cost. The detailed results of the moderated mediating analysis can be found in Table 2.

**TABLE 2:** Moderated mediating analysis from Studies 3a and 3b.

	Cost salience	Path	*B*	*SE*	*t*	*p*
Study 3a	Salient	Maximizing tendency → Perceived importance	4.72	1.95	2.42	0.017
		Perceived importance → Preference for large assortment	0.75	0.16	4.82	< 0.001
		Maximizing tendency → Preference for large assortment (total effect without mediator)	8.69	3.73	2.33	0.022
		Maximizing tendency → Preference for large assortment (direct effect with mediator)	5.15	3.53	1.46	0.146
	Not-salient	Maximizing tendency → Perceived importance	4.69	2.27	2.06	0.041
		Perceived importance → Preference for large assortment	0.25	0.18	1.41	0.161
		Maximizing tendency → Preference for large assortment (total effect without mediator)	5.86	4.24	1.38	0.170
		Maximizing tendency → Preference for large assortment (direct effect with mediator)	4.69	4.30	1.09	0.278
Study 3b	Salient	Maximizing tendency → Perceived importance	13.60	1.97	6.88	< 0.001
		Perceived importance → Preference for large assortment	0.40	0.12	3.28	0.001
		Maximizing tendency → Preference for large assortment (total effect without mediator)	16.80	2.80	6.01	< 0.001
		Maximizing tendency → Preference for large assortment (direct effect with mediator)	11.32	3.17	3.57	0.001
	Not-salient	Maximizing tendency → Perceived importance	12.89	1.87	6.88	< 0.001
		Perceived importance → Preference for large assortment	0.05	0.12	0.41	0.683
		Maximizing tendency → Preference for large assortment (total effect without mediator)	5.09	2.41	2.11	0.037
		Maximizing tendency → Preference for large assortment (direct effect with mediator)	4.47	2.84	1.57	0.110

## Study 3b

Study 3b was a replication of Study 3a, with two exceptions. First, the maximizing tendency was measured by the maximizing tendency scale, another widely used scale for measuring the maximizing tendency (Diab et al., 2008; Cheek and Goebel, 2020). Second, in the cost non-salience condition, the participants were explicitly told that the distance between their home and the two stores was the same.

### Method

#### Participants

Recruitment was open to 250 China-based participants from Credamo in exchange for a small payment (163 females, 87 males, *M*_*age*_ = 29.78 years, *SD* = 7.57).

#### Procedure and Materials

The procedures were similar to Study 3a. Participants first completed the maximizing tendency scale (Diab et al., 2008, Cronbach’s α = 0.88). After a short filter task, they read a scenario about buying a sweater and were asked, when making the decision, how important a sweater was to them on a 100-point scale. The scale was anchored from not important at all (= 0) to very important (= 100). Participants were then randomly assigned to the cost salient group and the cost non-salient group. In the cost salient group, participants were then given two stores identical to Study 2. In the cost non-salient group, the two stores only differed in the number of products, and the participants were told that the distance between their homes and the two stores are the same. The participants in both groups were instructed to indicate their relative preferences on a 100-point scale anchored at the preference for Store A (= 0) and the preference for Store B (= 100).

### Results and Discussion

#### Perceived Importance

Similar to Study 3a, the results of linear regression revealed that participants’ maximizing tendency positively predicted their perceived importance, *R*^2^ = 0.28, *B* = 13.26, *SE* = 1.34, β = 0.53, *t* (248) = 9.86, *p* < 0.001. Maximizers perceived the decisions as more important than satisficers.

#### Preference for a Large Assortment

Two liner regressions were conducted to test the main effect of maximizing tendency or perceived importance on preference for large assortments. Similar to Study 3a, participants’ maximizing tendency positively predicted their preference for large assortments, *R*^2^ = 0.09, *B* = 9.60, *SE* = 1.97, β = 0.30, *t* (248) = 5.01, *p* < 0.001. Participants’ perceived importance also positively predicted their preference for large assortments, *R*^2^ = 0.08, *B* = 0.36, *SE* = 0.07, β = 0.29, *t* (248) = 4.69, *p* < 0.001. We also examined the moderating role of cost salience (i.e., cost salient vs. cost not salient) on the relationship between perceived importance and preference for the large assortments, using the PROCESS macro for Model 1 (Hayes, 2013). The results revealed a significant cost salience—perceived importance interaction effect on preference, *F*(1,246) = 11.03, *p* = 0.001. Perceived importance positively predicted participants’ preference for the large assortments when more alternatives came with a salient cost (*R*^2^ = 0.22, *B* = 0.63, *SE* = 0.11, β = 0.46, *t* (123) = 5.81, *p* < 0.001), whereas perceived importance did not significantly predict participants’ preference for a large assortment when more alternatives came with no salient cost (*R*^2^ = 0.13, *B* = 0.14, *SE* = 0.10, β = 0.13, *t* (123) = 1.45, *p* = 0.150).

#### Moderated Mediation Analysis

We further tested a moderated mediation model using PROCESS Model 14 (Hayes, 2013), with maximizing tendency as the independent variable, preference as the dependent variable, perceived importance as a mediator, and cost as the moderator. A bootstrapping analysis with 5,000 iterations revealed that perceived importance mediated the relationship between maximizing tendency and preference under cost-salient conditions, as the 95% bias-corrected confidence interval for the indirect effect did not include zero [3.027, 10.321]. However, the mediating effect diminished under the cost non-salient condition, and the indirect effect included zero [–3.655, 2.879]. The detailed results of the moderated mediating analysis can be found in Table 2. Overall, the results of Study 3a were replicated.

## General Discussion

In this study, we examined how maximizers and satisficers perceived the importance of decisions differently, used perceived importance as a mediator, and tested the moderating role of the cost of large assortments. Study 1 provided basic evidence regarding whether maximizers perceive decisions as more important than satisficers. Study 2 demonstrated that perceived importance mediates the relationship between maximizing and preference for large assortments. Studies 3a and 3b found that decision-makers who perceive the decision as more important have a greater preference for large assortments only when the large assortment comes with a salient cost.

This research makes significant contributions to the literature on enhancing the understanding of maximizing decision-making. First, over the past 20 years, a tendency to search for more alternatives has been treated as a key characteristic of maximizers (Schwartz et al., 2002; Dar-Nimrod et al., 2009; Cheek and Schwartz, 2016; Cheek and Goebel, 2020). Building beyond the scope of existing research, we examine how perceived importance drives maximizers’ preference for large assortments. Second, while most previous research focuses on the preference (Weaver et al., 2015; Luan and Li, 2017), behaviors (Iyengar et al., 2006; Shiner, 2015; Goldsmith et al., 2018; Olson and Ahluwalia, 2021) and post-decision subjective feelings (Ma and Roese, 2014; Hassan et al., 2019) of maximizers and satisficers, we find that the difference between maximizers and satisficers occurs at an early stage and can sufficiently influence the subsequent stages.

Apart from the theoretical contributions, this research also has significant practical implications for the development of marketing strategies. Past research provides insight to marketers on pleasing maximizers (Chowdhury et al., 2009; Kokkoris, 2018; Khare et al., 2021); however, identifying maximizers in the marketplace is not always easy. According to this study, perceived importance can be used to increase the attractiveness of large or small retailers. For example, large retailers can adopt campaigns like “every decision matters” to increase consumers’ preference for large assortments without identifying whether the consumer is a maximizer or a satisficer. In addition, as the results of Studies 3a and 3b revealed the moderating role of cost salience, retailers with large assortments should avoid reminding consumers about cost information. Retailers could also try to decrease the cost by developing e-commerce, which may maintain their attractiveness to both maximizers and satisficers.

The limitations of this study should be acknowledged. First, this study used hypothetical situations. Although hypothetical situations are widely used in the maximizing literature (e.g., Weaver et al., 2015; Mao, 2016; Kokkoris, 2019), future research should examine whether the same results can be obtained by further increasing the ecological validity, for example, in real-life situations. Second, maximizers are measured rather than manipulated in this study. Future research can manipulate maximizing tendency (e.g., priming the maximizing mindset; Ma and Roese, 2014) to build a causal relationship between maximizing and perceived importance.

This study also presents several avenues for future work. First, as mentioned in the theoretical background, the choice of assortment can be conceptualized in two stages (Broniarczyk, 2008; Goodman and Malkoc, 2012). This study concentrates on exploring store individuals’ willingness to visit but does not explore how they search and compare the options inside a store. Future research could test whether this effect can be replicated in the second stage. Second, while this study focuses on the preference for large assortments, future research can go a step further to tap into the negative consequences of large assortments. A large stream of research found that while maximizers prefer a large assortment, it also makes them feel less satisfied and more regretful about what they have chosen (Carrillat et al., 2011; Besharat et al., 2014; Kokkoris, 2018; Hassan et al., 2019). Future research could explore whether perceived importance explains maximizers’ dissatisfaction and regret. Another related question is why maximizers think their decisions are more important. This study did not provide evidence interpreting this question, but future research can try to explore it in order to deepen the understanding of the findings of this research.

## Data Availability Statement

The raw data supporting the conclusions of this article will be made available by the authors, without undue reservation.

## Ethics Statement

The studies involving human participants were reviewed and approved by Department of Psychology, Tsinghua University. The patients/participants provided their written informed consent to participate in this study.

## Author Contributions

ML and ZL developed the study concept. ML and HL drafted the manuscript. ZL provided critical revisions. All authors contributed to the study design, data collection, analysis, and interpretation, and approved the final version of the document for submission.

## Conflict of Interest

The authors declare that the research was conducted in the absence of any commercial or financial relationships that could be construed as a potential conflict of interest.

## Publisher’s Note

All claims expressed in this article are solely those of the authors and do not necessarily represent those of their affiliated organizations, or those of the publisher, the editors and the reviewers. Any product that may be evaluated in this article, or claim that may be made by its manufacturer, is not guaranteed or endorsed by the publisher.
