# Supplementary material for: Taking Decisions Too Seriously: Why Maximizers Often Get Mired in Choices
Source: Front Psychol. 2022 Jun 15;13:878552. doi: 10.3389/fpsyg.2022.878552 (PMC9240276; doi:10.3389/fpsyg.2022.878552)
Supplement: Supplementary file 1 [file Data_Sheet_1.PDF]

Taking decisions too seriously: Why maximizers often get mired in choices

Methodology Reporting

1 Maximization Scale (Schwartz et al., 2002; Studies 1-3a)

1) When I watch TV, I channel surf, often scanning through the available options even while attempting to watch one program.
2) When I am in the car listening to the radio, I often check other stations to see if something better is playing, even if I'm relatively satisfied with what I'm listening to.
3) I treat relationships like clothing: I expect to try a lot on before I get to the perfect fit.
4) No matter how satisfied I am with my job, it's only right for me to be on the looked out for better opportunities.
5) I often fantasize about living in ways that are quite different from my actual life.
6) I'm a big fan of lists that attempt to rank things (the best movies, the best singers, the best athletes, the best novels, etc.).
7) I often find it difficult to shop for a gift for a friend.
8) When shopping, I have a hard time finding clothing that I really love.
9) Renting videos is really difficult. I'm always struggling to pick the best one.
10) I find that writing is very difficult, even if it's just writing a letter to a friend, because it's so hard to word things just right. I often do several drafts of even simple things.
11) No matter what I do, I have the highest standards for myself.
12) I never settle for second best.
13) Whenever I'm faced with a choice, I try to imagine what all the other possibilities are, even ones that aren't present at the moment.

Strongly disagree…… Strongly agree (1-7)

2 Maximizing Tendency Scale (Diab et al., 2008; Study 3b)

1) No matter what it takes, I always try to choose the best thing.
2) I don't like having to settle for "good enough."
3) I am a maximizer.
4) No matter what I do, I have the highest standards for myself.
5) I will wait for the best option, no matter how long it takes.
6) I never settle for second best.
7) I am uncomfortable making decisions before I know all of my options.
8) Whenever I'm faced with a choice, I try to imagine what all the other possibilities are, even ones that aren't present at the moment.
9) I never settle.

Strongly disagree…… Strongly agree (1-7)

3 Perceived Importance

Study 1

How important do you perceive buying a sweater [toothbrush / chocolate / smartphone / shoe / sunglasses / ice maker / shampoo / dish soap / pen / car] to be?

Study 2

How important do you perceive buying a sweater to be?

Study 3a

How important do you perceive buying a smartphone to be?

Study 3b

How important do you perceive buying a sweater to be?

Not important at all…… Very important (0-100)

4 Preference for large assortment

Study 2

Imagine that you are going to by a sweater, and you have the option of：

(A) walking to the nearest local store (5 minutes away) that offers 8 different types of sweaters

(B) driving to the shopping mall (40 minutes away) that offers 40 different types of sweaters.

Which store would you go to?

Store A…… Store B (0-100)

Study 3a - cost salient group

Imagine that you are going to by a smartphone, and you have the option of：

(A)walking to the nearest local store (5 minutes away) that offers 8 different smartphones.

(B) driving to the shopping mall (40 minutes away) that offers 40 different smartphones.

Which store would you go to?

Store A…… Store B (0-100)

Study 3a - cost not-salient group

Imagine that you are going to by a smartphone, and you have the option of：

(A) a store offers 8 different smartphones.

(B) a store offers 40 different smartphones.

Which store would you go to?

Store A…… Store B (0-100)

Study 3b - cost salient group

Imagine that you are going to by a sweater, and you have the option of：

(A) walking to the nearest local store (5 minutes away) that offers 8 different types of sweaters

(B) driving to the shopping mall (40 minutes away) that offers 40 different types of sweaters.

Which store would you go to?

Store A…… Store B (0-100)

Study 3b - cost not-salient group

Imagine that you are going to by a sweater, and you have the option of：

(A) a store offers 8 different types of sweaters

(B) a store offers 40 different types of sweaters

The distance between your home and the two stores are the same. Which store would you go to?

Store A…… Store B (0-100)
